# Endoscopic meatotomy in the treatment of ureterocele: results in adult patients

**DOI:** 10.11604/pamj.2020.36.243.24941

**Published:** 2020-08-04

**Authors:** Amine Oueslati, Ahmed Saadi, Marouene Chakroun, Selim Zaghbib, Abderrazak Bouzouita, Amine Derouiche, Mohamed Riadh Ben Slama, Haroun Ayed, Mohamed Chebil

**Affiliations:** 1 Urology Department, Charles Nicolle Hospital, Tunis, Tunisia

**Keywords:** Ureterocele, adult, endoscopy, meatotomy

## Abstract

To evaluate the efficacy of endoscopic meatotomy in the treatment of ureterocele in adults. A retrospective study of adult patients with ureterocele, treated between January 1987 and December 2014. In 47 patients, 55 intravesical ureteroceles were diagnosed and classified as 18 right, 21 left and eight bilateral (38%, 44% and 17% respectively). According to the Bruézière classification, 41 (75%) ureteroceles were type A and 14 (25%) others were type C. These ureteroceles were complicated by calculus formation in 22 cases, moderately dilated excretory pathways in 16 cases and both complications in a total of 9 cases. Four patients had a complicated ureterocele with pyelonephritis, one of which was emphysematous. The endoscopic treatment was performed in cases of complicated and/or symptomatic ureteroceles. Fifty one cases were treated by a “smiling mouth” meatotomy consisting in a transverse horizontal incision, with the treatment of any associated complication. The mean operative time was 35 minutes (10-90). The operative follow-up was uneventful in 42 patients and complications occurred in 5 patients (2 urinary retentions, 2 infectious complications and one hematuria). The mean duration of postoperative stay was 1-2 days. The mean follow-up was 15 months. Four patients developed vesicoureteral reflux and no stenosis was noted. The endoscopic incision of the ureteroceles seems today, after reviewing the results, to be a good treatment of adult ureterocele. It is a simple, minimally invasive and has a low morbidity rate.

## Introduction

Ureterocele is a rare congenital malformation characterized by pseudo-cystic dilation of the lower extremity of the ureter [1]. It is often discovered in neonates on antenatal ultrasonography which explains its rarity in adults and few publications concerning adult ureterocele. Its treatment is not unequivocal; it is indicated in the case of complicated or symptomatic ureterocele. In order to evaluate short and long term results of endoscopic meatotomy, we proposed a retrospective study of 47 adult patients with endoscopically treated ureterocele.

## Methods

**Figure 1:**
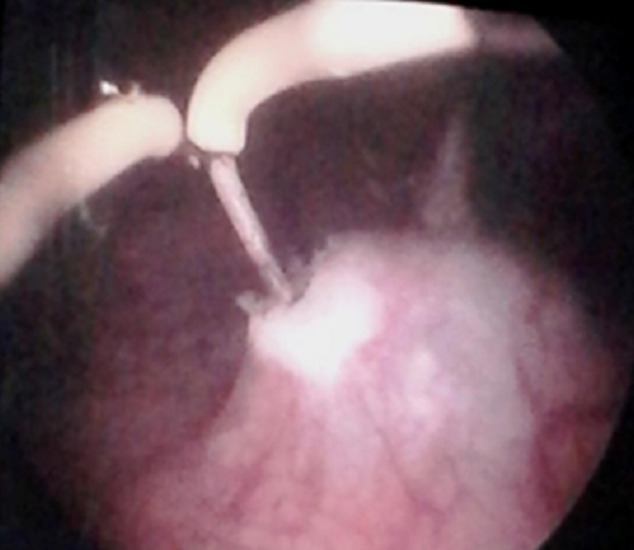
"smiling mouth" meatotomy: intraoperative image

This is a retrospective study of 47 cases of patients treated for ureterocele between January 1987 and December 2014 in our department. We included in this study: patients aged 16 years old or older; symptomatic ureteroceles; ureteroceles associated with at least one complication among the following: repercussion on the upper urinary tract, calculus formation and infection. A renal function test and cytobacteriological examination of urine were performed on all patients. Radiological investigations consisted of intravenous urography or computes tomography urography in all patients. Renal ultrasound was performed in patients presenting a dilatation of the upper apparatus before surgery, to investigate the quality and thickness of the renal parenchyma. The classifications of ureterocele by Bruezière [1] into four types was adopted: type A: intra-vesical ureterocele on a single ureter; type B: ectopic ureterocele on single ureter; type C: intra-vesical ureterocele on pyelo-ureteral duplicity; type D: ectopic ureterocele on pyelo-ureteral duplicity. We performed the surgical technique described by Rodriguez [2]: the bladder being in semi-repletion, a semicircular incision is made at the base of the ureterocele, extending 3 to 8mm on each side of the ureteral meatus (Figure 1). Postoperative monitoring was clinical and radiological. The control of the upper urinary tract was performed either by an intravenous urography, computed tomography urography or by renal ultrasound. A retrograde urethrocystography for vesicoureteral reflux was performed in only 30 patients.

## Results

**Figure 2:**
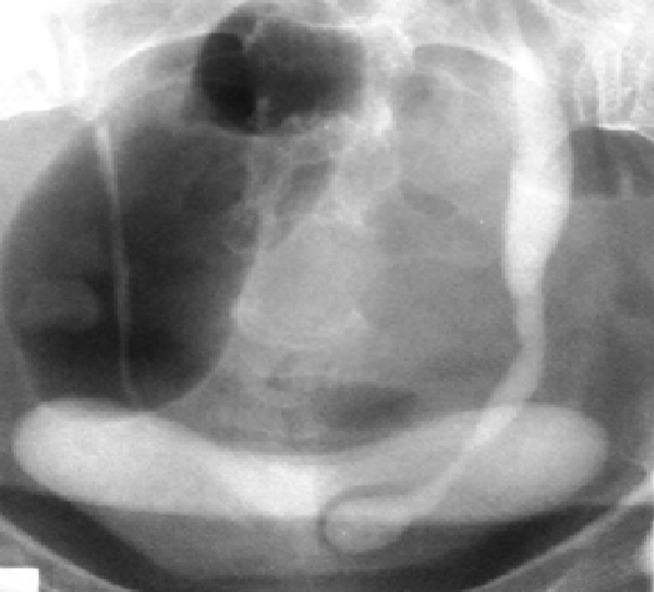
early snapshot of intravenous urography: image of “snake head” surrounded by a light halo on the cystogram

**Figure 3:**
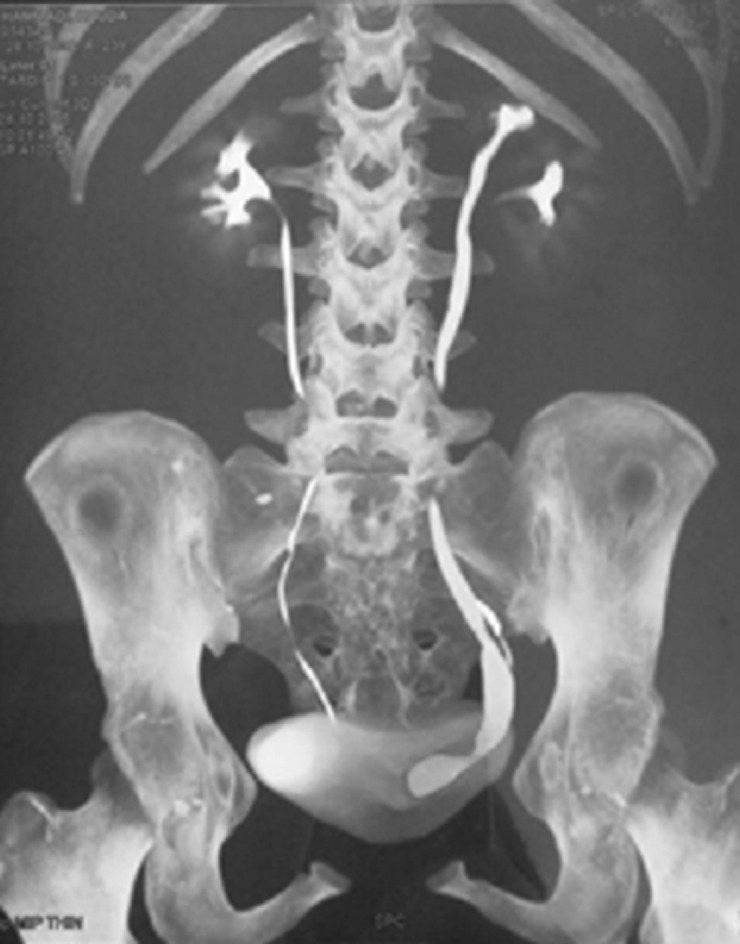
CT urography reconstruction layout of left ureterocele with left duplicated ureter at the expense of the upper renal lobule

**Figure 4:**
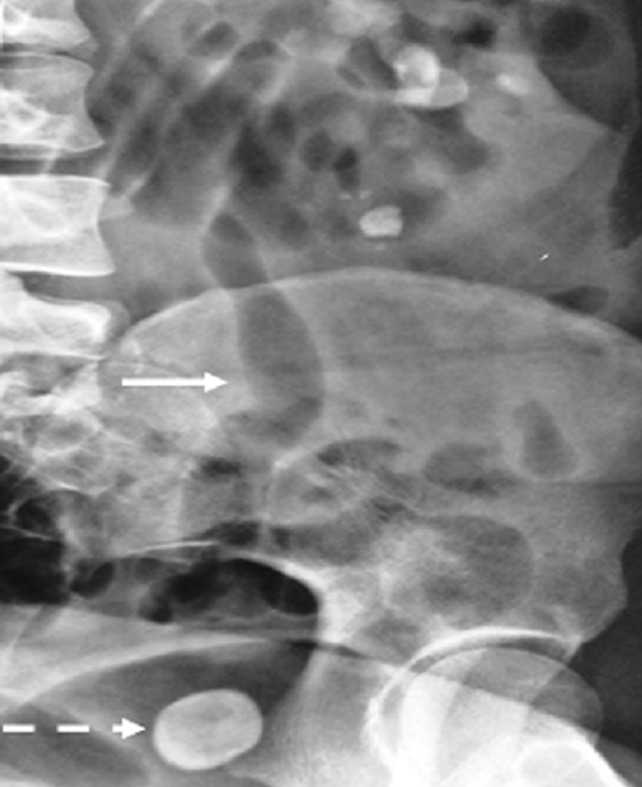
abdominal radiography of emphysematous pyelonephritis complicating ureterocele; full arrow: airy light in the left kidney and ureter; dotted arrow: ureteral pelvic lithiasis complicating ureterocele

The average age of our population was 42 years old (19-71). The sex predominance was female with a sex ratio of 1: 3. The symptoms were dominated by a low back pain in 66% of cases (Table 1). An intravenous urography or computes tomography urography was performed in all patients to diagnose ureterocele in 88.5% of cases by showing the classic appearance of the terminal ureter as a “snake head” (Figure 2, Figure 3). The remaining cases were first diagnosed with a pelvic ureteral stone on preoperative imaging and the diagnosis of ureterocele was made operatively. In the 47 patients, 55 intravesical ureteroceles were diagnosed in 47 patients as follows: eighteen on the right side, 21 on the left side and eight ureteroceles were bilateral (38%, 44% and 17% respectively). All ureteroceles were intravesical and according to the Bruézière classification, 41 (75%) ureteroceles were type A and 14 (25%) ureteroceles were type C. Their size ranged from 1 to 4cm. The renal parenchyma was preserved in all cases. In situ stone was found in 22 cases, moderate dilation of the excretory pathways was found in 16 cases and 9 cases had both complications. Four patients presented pyelonephritis, one of which was emphysematous (Figure 4). Four cases of ureteroceles were discovered fortuitously in four patients with symptomatic contralateral ureteroceles, they were centimetric and uncomplicated, justifying therapeutic abstention.

**Table 1:** clinical symptomatology of ureterocele in our patients

Symptoms	Number of Patients	Rate (%)
Low Back Pain	31	66
Mouth Disorders	25	53
Renal Colic	12	25
Hematuria	11	23
Asymptomatic	2	4

An endoscopic meatotomy was performed in cases of complicated and/or symptomatic ureteroceles (51 cases). The mean operating time was 35 minutes (10-90), taking into account the treatment of an eventual calculus. In the case of stone formation, meatotomy allowed stone elimination and in situ lithotripsy procedure was necessary for larger calculi. The mean size of the calculus was 1.7cm (0.5-3). A 24 hours trans-urethral bladder drainage by a Foley 16 catheter probe was performed in 32 patients. No surgical incidents were noted. The average hospital stay was 1-2 days and follow-up was uneventful in 42 patients. In the remaining patients, we noted an acute urinary retention in 2 patients, hematuria in 1 patient and a post-operative fever in another patient. All of our early post-operative complications were grade 1 according to the classification of Clavien. The patient who had emphysematous pyelonephritis evolved poorly after endoscopic meatotomy and ureteral stenting and a secondary nephrectomy was required. The mean follow-up was about 15 months. Four patients developed vesicoureteral reflux, with a grade IV reflux in two cases, requiring a nephroureterectomy for a destroyed kidney in one case and ureteral reimplantation in the other case. In the other two cases, vesicoureteral reflux disappeared within 6 months. No stenosis was noted.

## Discussion

Ureterocele is a cystic dilatation of the sub-mucosal segment of the intravesical ureter, and its etiology remains uncertain [1,3]. This is an entity commonly observed in newborns and children, most often accompanied by ureteric duplicity and ectopic abortion of the ureteral meatus in the urethra [3]. Adult ureteroceles rarely occur on duplicity and are exceptionally extra vesical [4]. In our series, 75% of the ureteroceles were classified as intravesical on a single ureter (type A according to the Bruézière classification). This high frequency of simple forms explains the good tolerance and often moderate repercussions of adult ureteroceles [3]. Treatment, indicated in complicated or symptomatic cases, is not univocal [4]. The treatment aims to prevent urinary tract infections, preserve renal function, ensure good ureterovesical drainage, prevent vesicoureteral reflux, to minimize surgical complications with a minimum number of procedures and to retain the possibility of further reconstruction [3-5]. The evolution of endo-urological techniques and the better understanding of the anti-reflux mechanisms of the ureter has resulted in a renewed interest in endoscopic meatotomy, which is increasingly being practiced.

The technique gradually evolved towards a shorter, more pronounced incision retaining the detrusor muscle posterior wall and respecting the valve mechanism ensured by the inert mucosa that relaxes under the pressure of the filling bladder [2]. It is the principle of the endoscopic meatotomy “smiling mouth” technique described by Rodriguez [2]. The endoscopic meatotomy according to Rodriguez was also performed using a laser type Nd-YAG by Gupta *et al*. [6] and type Ho-YAG by Mazo *et al*. [7] with good results. Liu *et al*. treated ureteroceles in 40 adults by selective laser vaporization of the greenlight type and concluded that this technique is safe and effective for the treatment of orthotopic ureterocele in adults [3]. The comparison between different endoscopic techniques is difficult because there is no prospective, randomized, controlled study. Therefore, accurate statistical analysis between methods is impossible. Endoscopic meatotomy is recommended as a first-line treatment for intravesical ureterocele on a single ureter in adults and it is the final treatment in 77% to 93% of cases [8,9]. This meatotomy can be performed in cases of ureteric duplicity without difficulty [4,10] which was the case in 14 of our patients.

In these cases, a reflux involving the ureter of the lower segment may disappear spontaneously in 30% of cases due to the elimination of a possible cervical obstruction and the retraction of the ureterocele. However, the efficacy of endoscopic treatment of ectopic ureteroceles has not been proved and this approach is not generally considered a definitive treatment for these types of ureteroceles [11]. Some authors have proposed first endoscopic treatment for children with ectopic ureteroceles because the early decompression of the ureterocele permits preservation of the upper urinary tract and facilitates the final surgical treatment (removal of the ureterocele followed by ureterovesical re-implantation). Endoscopic treatment leaves the pouch of the ureterocele in place and the course may be marked by the appearance of vesicoureteral reflux or stenosis. The vesicoureteral reflux should be systematically investigated after 3 to 6 months of surgical procedure. The size of the ureterocele (greater than 3cm), its ectopic position and the dilatation of the lower ureter are the three main predictors of this complication [12]. Vesicoureteral reflux post-meatotomy does not mean failure of the treatment because the evolution of this reflux is often favorable. Besides, endoscopic treatment does not cut bridges to ureterovesical re-implantation in the event of symptomatic or complicated reflux. In the case of stenosis, a new meatotomy can be attempted [11].

## Conclusion

The endoscopic incision of ureteroceles seems today, after reviewing its results, to be the first choice of treatment for adults with complicated or symptomatic ureteroceles. It is a simple, minimally invasive and low-morbidity technique that perfectly meets the objectives of ureterocele treatment. However, the risk of vesicoureteral reflux or stenosis requires a strict follow-up.

### What is known about this topic

- Ureterocele is a rare congenital malformation;
- It´s usually diagnosed in children and its discovery is uncommon in adults;
- Treatment of ureterocele in adults is not codified.

### What this study adds

- We described ureterocele endoscopic management in 47 adults which is a considerable population;
- Endoscopic treatment of adult symptomatic or complicated ureterocele seems to be “a treatment of choice” due to its simplicity and efficacy with few complications.
